# Editorial: Limbic Encephalitis: Autoimmune Impact on Inhibitory GABAergic Neuronal Pathways in Temporal Lobe Epilepsy

**DOI:** 10.3389/fneur.2016.00036

**Published:** 2016-03-17

**Authors:** Nico Melzer, Christian Geis, Sven G. Meuth

**Affiliations:** ^1^Department of Neurology, University of Münster, Münster, Germany; ^2^Department of Neurology, Jena University Hospital, Jena, Germany

**Keywords:** epilepsy, temporal lobe, limbic system, encephalitis, memory disorders, seizures, T cells, B cells

**The Editorial on the Research Topic**

**Limbic Encephalitis: Autoimmune Impact on Inhibitory GABAergic Neuronal Pathways in Temporal Lobe Epilepsy**

Mesial temporal lobe epilepsy (mTLE), a common adult epilepsy syndrome, is generally acquired. Recent data have demonstrated autoimmune inflammation predominantly affecting the limbic structures of the brain as a cause of adult mTLE development (1–3). Patients usually present with mesial temporal lobe seizures with interictal temporal epileptiform activity and slowing, episodic memory disturbance, and a variety of other behavioral, emotional, and cognitive changes. Magnetic resonance imaging typically exhibits volume and signal changes of the amygdala and hippocampus, and specific anti-neuronal antibodies binding to either intracellular or plasma membrane neuronal antigens can be detected in sera and cerebrospinal fluid.

Glutamic acid decarboxylase (GAD) (4) and γ-aminobutyric acid (GABA)-B receptors (5) have recently been identified as distinct neuronal antigens in limbic encephalitis. This suggests a possible involvement of disturbed inhibitory GABAergic signaling in the etiology of seizures and neuropsychiatric symptoms characterizing this form of autoimmune epilepsy.

Due to restricted access to their target antigen, autoantibodies directed toward intracellular GAD are believed to bear limited pathogenic potential, and neurons may be affected by autoreactive T cells instead (1). In contrast, autoantibodies against plasma membrane GABA-B receptors have been suggested to exert direct pathogenic effects (6), and the role of autoreactive T cells in these disorders is unclear at present (1).

In the Frontiers in Neurology Research Topic “Limbic Encephalitis: Autoimmune Impact on Inhibitory GABAergic Neuronal Pathways in Temporal Lobe Epilepsy,” authors provide a survey on clinical and scientific aspects of autoimmune inflammation in mTLE.

Ehling et al. review and discuss the role of CD8^+^ T cells–neuron interactions, whereas Seebohm et al. summarize the current knowledge and discuss technical approaches to study the autoantibody–receptor interactions in limbic encephalitis.

Haselmann et al. present a methodical approach to investigate the impact of stereotactically injected human IgG fractions on GABAergic signaling in intact murine hippocampal network *ex vivo*. Stemmler et al. could not detect any effect of serum of a patient with confirmed GAD antibody-associated limbic encephalitis on GABAergic neurotransmission in murine cultured hippocampal networks, challenging the view that the presence of such autoantibodies compromise inhibitory network function. Widman et al. report a correlation of the CD8^+^ T cells in the cerebrospinal fluid with clinical and paraclinical measures of disease activity together with an unambiguous response to treatment with basiliximab, a chimeric mouse–human monoclonal antibody to the α-chain of the interleukin-2 receptor (CD25) on T cells in a patient with GAD antibody-associated limbic encephalitis. This strongly argues in favor of a pathogenic role of CD8^+^ T cells in this form of autoimmune temporal lobe epilepsy.

The amygdala is central for the generation of adequate homoeostatic behavioral responses to emotionally significant external stimuli following processing in a variety of parallel neuronal circuits. Melzer et al. hypothesize that adaptive cellular and humoral autoimmunity may target and modulate distinct inhibitory or excitatory neuronal networks within the amygdala and thereby strongly impact processing of emotional stimuli and corresponding behavioral responses in patients with limbic encephalitis. Indeed, Schroder et al. show defective modulation of sympathetic ­autonomic responses during emotional stimulation in limbic encephalitis probably due to impaired functioning of the amygdala. Witt et al. present a case of GAD antibody-associated limbic encephalitis with predominant involvement of the left amygdala that presented with retrograde episodic memory impairment, characterized by loss of emotional attachment and autonoetic awareness of usually highly emotional autobiographical memories consistent with the known role of the amygdala as part of the basolateral limbic circuit relevant for emotional valence of memories. Furthermore, a special form of anterograde episodic memory impairment was present called accelerated long-term forgetting, consisting of accelerated loss or impaired access to newly encoded episodes. These cognitive and emotional disturbances occurred in the absence of overt epileptic seizures, suggesting a direct causal role of autoimmune inflammation within temporomesial structures.

## Author Contributions

NM, CG, and SM contributed equally to this work. All authors approved the final version of the manuscript.

## Conflict of Interest Statement

The authors declare that the research was conducted in the absence of any commercial or financial relationships that could be construed as a potential conflict of interest.
